# Ca^2+^-Activated Ion Channels Exert Opposite Effects in Different Signaling Compartments of Vomeronasal Sensory Neurons

**DOI:** 10.1523/JNEUROSCI.2134-24.2025

**Published:** 2025-03-03

**Authors:** Rudolf Degen, Victoria K. Switacz, Jennifer Spehr, Marc Spehr

**Affiliations:** ^1^Department of Chemosensation, Institute for Biology II, RWTH Aachen University, Aachen 52074, Germany; ^2^Research Training Group 2416 MultiSenses–MultiScales, RWTH Aachen University, Aachen 52074, Germany

**Keywords:** chemosensory, ion channels, olfaction, physiology, sensory neurons, vomeronasal organ

## Abstract

In most mammals, conspecific chemical cues that drive innate social and sexual behavior are detected by the vomeronasal organ (VNO) and processed in the accessory olfactory bulb (AOB). Chemosensory stimulation of vomeronasal sensory neurons (VSNs) at their microvillous dendritic knobs triggers, first, a local signal transduction and amplification cascade and, second, transformation of that signal into action potential (AP) discharge at the soma. Both processes—signal transduction and AP generation—involve local Ca^2+^ elevations in the knob and soma, respectively. Here, we revisit the somewhat still controversial functions of Ca^2+^-activated ion channels in both VSN compartments. In acute mouse VNO slices (of either sex), focal photorelease of Ca^2+^ reveals that VSN knob and soma both act as independent Ca^2+^ signaling compartments, in which Ca^2+^ elevations exert opposite effects. While Ca^2+^ signals in the knob drive an excitatory inward current, Ca^2+^ elevations in the soma primarily activate hyperpolarizing outward currents that silence VSNs. A substantial fraction of the latter current is mediated by SK and/or BK channels. Notably, SK channel activity strongly affects VSN firing. Together, our study reveals a diverse composition of Ca^2+^-activated currents in VSN somata and uncovers an unexpected role of SK channels in dampening excitability and, thus, in controlling VSN-to-AOB information transfer.

## Significance Statement

Cytosolic Ca^2+^ signals play an important role in vomeronasal neuron function. Both sensory signal transduction and information transfer via action potentials (APs) involve transient Ca^2+^ elevations. Using local Ca^2+^ uncaging during single-cell electrophysiological recordings, we demonstrate that Ca^2+^-activated ion channels exert opposite functions during primary transduction versus AP firing. Specifically, SK channels are primarily involved in dampening vomeronasal firing.

## Introduction

Conspecific chemical communication controls social and sexual behavior. In most mammals, rodents in particular, behaviorally instructive chemosignals are detected and processed by the accessory olfactory system. Its peripheral sensory structure, the vomeronasal organ (VNO), is a paired cylindrical organ located at the anterior base of the nasal septum. Each of the VNO's two blind-ended tubes harbors ∼200,000 vomeronasal sensory neurons (VSNs) in a crescent-shaped pseudostratified neuroepithelium (Jacobson et al., 1998; Mohrhardt et al., 2018). Each bipolar VSN extends a single unbranched dendrite that terminates in a microvillous swelling (knob), which is immersed in the mucus of the VNO lumen (Tirindelli et al., 2009). From their basal pole, VSNs project a long unmyelinated axon that targets the glomerular layer of the accessory olfactory bulb (AOB; Meredith, 1991; Rodriguez et al., 1999), the first central processing stage along the accessory olfactory pathway.

VSNs detect and transduce chemosignals at their microvillous dendritic knobs. As primary sensory neurons, VSNs then transform suprathreshold transduction currents into action potential (AP) discharge that is relayed to the brain. Both types of sensory events, signal transduction and transformation, involve substantial cytosolic Ca^2+^ elevations within VSN knobs and somata, respectively. The primary signal transduction cascade is based on phospholipid turnover and culminates in successive opening of TRPC2 (Liman et al., 1999) and TMEM16A (Amjad et al., 2015) channels. The TRPC2 channel serves an important function, though TRPC2-independent vomeronasal signals have also been reported (Kelliher et al., 2006; Yu, 2015). TRPC2-mediated Ca^2+^ influx (Lucas et al., 2003) fuels both Ca^2+^/calmodulin-dependent channel inhibition (Spehr et al., 2009) and TMEM16A-dependent signal amplification (Yang and Delay, 2010; Kim et al., 2011; Dibattista et al., 2012; Amjad et al., 2015; Münch et al., 2018). A substantially elevated Cl^−^ level in VSN dendrites (Kim et al., 2015; Untiet et al., 2016) provides the electrochemical driving force necessary for boosting sensory responses via TMEM16A-mediated Ca^2+^–activated Cl^−^ currents. Secondary phospholipid metabolites such as arachidonic acid (Spehr et al., 2002; Zhang et al., 2010) likely play a modulatory role. Previously, the small-conductance Ca^2+^–sensitive K^+^ channel (SK3) was proposed to serve as an alternative route for VSN activation (Kim et al., 2012). Mice with a global *Kcnn3* deletion display altered mating behaviors and aggression phenotypes. While intriguing, the global nature of the deletion complicates interpretation of the behavioral effects (Mohrhardt et al., 2018).

In VSN somata, Ca^2+^ entry via voltage-gated channels is tightly coupled to stimulus-evoked AP discharge. Therefore, such Ca^2+^ elevations have frequently been used as a proxy for VSN activity (Leinders-Zufall et al., 2000, 2004; Spehr et al., 2002; Chamero et al., 2007; Haga et al., 2007, 2010; Nodari et al., 2008; Papes et al., 2010; Lê Cao et al., 2011; Turaga and Holy, 2012; Wong et al., 2018; Nagel et al., 2024). Both low voltage-activated T–type and high voltage-activated L-, N-, and P/Q–type Ca^2+^ channels (Liman and Corey, 1996; Fieni et al., 2003; Ackels et al., 2014) link VSN firing to perinuclear Ca^2+^ signals, which in turn may modulate VSN firing (Ukhanov et al., 2007). Here, coupling of Ca^2+^-sensitive large–conductance K^+^ (BK) channels (Marty, 1981) with L-type Ca^2+^ channels has been proposed as a requirement for persistent VSN firing (Ukhanov et al., 2007). In contrast, others suggested that BK channels have an opposite effect and play a role in arachidonic acid-dependent sensory adaptation (Zhang et al., 2008).

Both SK and BK are found in various (non)neuronal tissues. While depolarization and cytoplasmic Ca^2+^ elevations converge on BK channel gating (Berkefeld and Fakler, 2008), SK channels are activated solely by intracellular Ca^2+^ (Berkefeld et al., 2010). BK channel activation requires high local Ca^2+^ concentrations (∼10 µM) and thus depends on tight coupling to voltage-activated Ca^2+^ channels. SK activation, on the other hand, with EC_50_ values of ∼0.3 µM, is fueled by a variety of cellular Ca^2+^ sources (Berkefeld et al., 2010). While BK channels mediate the fast phase of afterhyperpolarization (Niday and Bean, 2021) and affect presynaptic release (Contreras et al., 2013), SK produces a slow afterhyperpolarization and shapes postsynaptic responses (Bond et al., 2005).

Here, we revisit the somewhat still controversial functions of Ca^2+^-activated ion channels in VSN knobs and, specifically, somata. Focal photorelease of Ca^2+^ in defined VSN compartments triggers distinct electrical signals. While transient Ca^2+^ elevations in the knob induce depolarizing inward currents (Dibattista et al., 2012), Ca^2+^ uncaging in the soma primarily triggers hyperpolarizing outward currents that silence VSNs. A substantial fraction of Ca^2+^-activated activity in VSN somata is mediated by SK and/or BK channels, though a distinct VSN subpopulation also expresses somatic Ca^2+^-activated Cl^−^ currents. Notably, SK channel activity strongly affects AP firing, whereas both BK-mediated and Ca^2+^-activated Cl^−^ currents exert only minor effects on VSN output. Together, our study reveals a diverse composition of Ca^2+^-activated currents in VSN somata and uncovers an unexpected role of SK channels in controlling VSN discharge.

## Materials and Methods

### Animals

All animal procedures were approved by local authorities and in compliance with both European Union legislation (Directive 2010/63/EU) and FELASA recommendations. All experimental procedures were approved by the State Agency for Nature, Environment and Consumer Protection (LANUV). Mice were housed in littermate groups separated by sexes [room temperature (RT); 12:12 h light/dark cycle; food and water available *ad libitum*]. Experiments used adult (12–32 weeks) C57BL/6J mice (Charles River Laboratories) of either sex.

### Chemicals and solutions

The following solutions were used:

(S_1.1_) 4-(2-Hydroxyethyl)piperazine-1-ethanesulfonic acid (HEPES) buffered extracellular solution containing (in mM) 145 NaCl, 5 KCl, 1 CaCl_2_, 1 MgCl_2_, 10 HEPES; pH 7.3 (adjusted with NaOH); osmolarity, 300 mOsm (adjusted with glucose).

(S_1.2_) Reduced Cl^−^ HEPES-buffered extracellular solution containing (in mM) 145 Na-d-gluconate, 5 K-d-gluconate, 1 CaCl_2_, 1 MgCl_2_, 10 HEPES; pH 7.3 (adjusted with NaOH); osmolarity, 300 mOsm (adjusted with glucose).

(S_2.1_) Oxygenated (95% O_2_, 5% CO_2_) extracellular solution containing (in mM) 120 NaCl, 25 NaHCO_3_, 5 KCl, 1 CaCl_2_, 1 MgSO_4_, 5 *N*,*N*-bis(2-hydroxyethyl)-2-aminoethanesulfonic acid (BES); pH 7.3 (adjusted with NaOH); osmolarity, 300 mOsm (adjusted with glucose).

(S_2.2_) Reduced Cl^−^ oxygenated (95% O_2_, 5% CO_2_) extracellular solution containing (in mM) 118 Na-d-gluconate, 25 NaHCO_3_, 5 K-d-gluconate, 2 NaCl, 1 CaCl_2_, 1 MgSO_4_, 5 BES; pH 7.3 (adjusted with NaOH); osmolarity, 300 mOsm (adjusted with glucose).

(S_3_) Standard pipette solution with VSN-specific [Cl^−^] containing (in mM) 93.5 K-gluconate, 42 KCl, 1 EGTA, 0.3 CaCl_2_, 10 HEPES, 2 Mg-ATP, 1 Na-GTP (free Ca^2+^, 106 nM); pH 7.1 (adjusted with KOH); osmolarity, 290 mOsm (adjusted with glucose).

(S_4_) Pipette solution for Ca^2+^ uncaging containing (in mM) 93.5 K-gluconate, 42 KCl, 2.5 *o*-NP-EGTA/10 K^+^, 2 CaCl_2_, 10 HEPES, 2 Mg-ATP, 1 Na-GTP (free Ca^2+^, 984 nM); pH 7.1 (adjusted with KOH); osmolarity, 290 mOsm (adjusted with glucose).

(S_5_) Cs^+^-based pipette solution for Ca^2+^ uncaging containing (in mM) 119.9 gluconic acid, 113.9 CsOH, 11.6 CsCl, 2.5 *o*-NP-EGTA/10 K^+^, 2 CaCl_2_, 10 HEPES, 2 Mg-ATP, 1 Na-GTP (free Ca^2+^, 984 nM); pH 7.1 (adjusted with CsOH); osmolarity, 290 mOsm (adjusted with glucose).

Free Ca^2+^ concentrations were calculated using WEBMAXCLITE v1.15 (RRID:SCR_000459); calculations based on 20°C, pH 7.1. Calculations for solutions including *o*-nitrophenyl ethylene glycol tetraacetic acid (*o*-NP-EGTA; obtained as a tetrapotassium salt) were performed using the EGTA calculator. If not stated otherwise, chemicals were purchased from Sigma-Aldrich. NP-EGTA was purchased from Thermo Fisher Scientific. Apamin and iberiotoxin were purchased from Abcam. Cal-520 was purchased from Biomol.

For focal stimulation and fast bath exchange, solutions and agents were applied from air pressure-driven reservoirs via an 8-in-1 Ø 250 μm multibarrel “perfusion pencil” (AutoMate Scientific). Changes in focal superfusion (Veitinger et al., 2011) were software-controlled and synchronized with data acquisition by TTL input to 12 V DC solenoid valves using a TIB 14S digital output trigger interface (HEKA Elektronik). Throughout experiments, constant flow (S_1.1_ or S_1.2_) was maintained to avoid mechanical/motion artifacts from switching valves. Both standard (S_1.1_ and S_2.1_) and reduced Cl^−^ (S_1.2_ and S_2.2_) solutions were applied simultaneously via both the bath and perfusion pencil. We routinely switched between control valves (e.g., S_1.1_ vs S_1.1_) during experiments to control for mechanical/motion artifacts.

### Slice preparation

Acute coronal VNO slices from adult mice were prepared as previously described (Hagendorf et al., 2009; Cichy et al., 2015) with minor modifications. Briefly, mice were killed by brief exposure to a CO_2_ atmosphere, cervical dislocation, and decapitation with sharp surgical scissors. The lower jaw, incisors, and soft palate were rapidly removed. The VNO was dissected, embedded in 5% low-gelling temperature agarose (dissolved in S_1.1_; Bio-Budget Technologies), and placed in ice-cold oxygenated S_2.1_, and coronal slices (150 μm) were cut on a VT1000S vibrating microtome (0.15 mm/s, 73 Hz; RRID:SCR_016495; Leica Biosystems). Slices were transferred to a submerged, chilled, and oxygenated storage chamber with circulating S_2.1_ until use.

### Electrophysiology

VNO slices were transferred to a recording chamber (Luigs & Neumann), positioned with stainless-steel anchors, and visualized using an upright fixed-stage video microscope (DM6000FS, Leica Microsystems) equipped for infrared-optimized differential interference contrast and epi-fluorescence imaging. Slices were continuously superfused with oxygenated S_2.1_ (∼1.5 ml/min, gravity flow). Neurons were visualized using a 25× (HCX IRAPO L25x/0.95W) and a 63× (HCX APO L U-V-I 63×/0.90 WI CS2) objective (Leica Microsystems), respectively. Patch pipettes (5–11 MΩ) were pulled from borosilicate glass capillaries (outer diameter, 1.50 mm; inner diameter, 1.0 mm; Science Products) on a PC-10 micropipette puller (Narishige Instruments), fire-polished (MF-830 Microforge, Narishige Instruments), and filled with pipette solution (S_3_–S_5_, depending on experimental design). An agar bridge (150 mM KCl) connected the reference electrode and bath solution. Data were acquired using an EPC-10 USB amplifier controlled by the Patchmaster 2 × 90.5 software (HEKA Elektronik). We monitored and compensated pipette and membrane capacitance as well as series resistance. Only neurons exhibiting stable access resistances and membrane resistances of ≥1 GΩ were used for analysis. Liquid junction potentials were calculated using the JPCalcW software (Barry, 1984) and corrected online. Signals were low-pass filtered [analog three- and four-pole Bessel filters (−3 dB); adjusted to ^1^/_3_–^1^/_5_ of the sampling rate (10–50 kHz)]. If not stated otherwise, holding potential (*V*_hold_) was between −70 and −75 mV. For current-clamp recordings, the injection current (*I*_inj_) was ≤−15 pA to maintain a membrane potential of approximately −75 mV. All pulse protocols (both voltage-clamp and current-clamp) are depicted as diagrams in figures and detailed in the corresponding captions. All electrophysiological data were recorded at RT. VSN membrane capacitance (*C*_mem_) was obtained immediately after membrane rupture using a square pulse (5 mV, 10 ms) routine. Original example current traces are depicted as absolute values, whereas average population data are plotted as current densities (pA/pF).

### Ca^2+^ uncaging

Photorelease of Ca^2+^ from *o*-NP-EGTA was triggered by focal illumination with a 375 nm diode laser (DL-375, Rapp OptoElectronic), controlled by the UGA42 Firefly point-scanning device for localized photomanipulation and SysCon software (v1.1.8.0; Rapp OptoElectronic). User-defined regions of interest (ROIs) were illuminated for 0.1 ‒ 3 s (7 µW/µm^2^ laser power density). ROIs were drawn to target distinct VSN compartments (soma, dendrite, or knob). For larger ROIs, point-scanning illumination entails that the total duration of scanning (i.e., “object time”) exceeds the illumination period per spot (i.e., “illumination time”). Here, object time is indicated in figures by violet bars, while illumination time is stated in seconds.

### Fluorescence Ca^2+^ imaging

Changes in VSN cytosolic Ca^2+^ concentration were routinely monitored by widefield fluorescence microscopy. VSNs were diffusion loaded via the patch pipette with the membrane-impermeable Ca^2+^–sensitive dye Cal-520. A custom filter cube (DCLP, 495 nm; EMBP, 535/70; Chroma Technology, Bellows Falls, VT) and an extra dichroic mirror (DCLP-423, Rapp OptoElectronic) allowed both 375 nm diode laser transmission and simultaneous Cal-520 excitation (X-Cite 200DC; Excelitas Technologies) and emission. Images were acquired by a scientific CMOS camera (Prime BSI; RRID:SCR_018464; Teledyne Photometrics) at a resolution of 1,024 × 1,024 pixels, 16 bit depth, and an acquisition rate of 9.1 fps. Each electrophysiological measurement was preceded by a 2 s recording of Ca^2+^ levels at −75 mV (*V*_hold_) to establish individual baselines (*F*_0_). Electrophysiological recordings, Ca^2+^ uncaging, and fluorescence Ca^2+^ imaging were triggered and synchronized using MetaMorph software (v7.10.2.240; RRID:SCR_002368; Molecular Devices).

### Experimental design and statistical analysis

All data were obtained from independent experiments performed on >3 d using >3 different animals. Individual numbers of experiments (*n*) are denoted in the respective figures and/or captions. If not stated otherwise, results are presented as either mean ± SD or median ± SD as indicated.

Statistical analyses were performed by, first, using Kolmogorov–Smirnov tests for normality and, second, using Wilcoxon signed rank tests, Mann–Whitney *U* tests, or paired students *t* tests (as dictated by data distribution and experimental design). Tests and corresponding *p* values that report statistical significance (≤0.05) are individually specified in captions. Data were analyzed offline using MATLAB v9.9/R2020b (RRID:SCR_001622; The MathWorks), Python scripts (Spyder IDE v5.5.3 in Anaconda Distribution, Python v3.9), functions in IGOR Pro 8.0.4 (RRID:SCR_000325; WaveMetrics), or Excel 2016 (v2102; Microsoft).

#### Electrophysiology

For isolation of Ca^2+^-activated currents from 30 consecutive downward voltage ramp recording segments, a baseline current–voltage relationship (*I*–*V* trace) was calculated by averaging *I*–*V* traces #4–#6 (i.e., before Ca^2+^ uncaging). This resulting baseline *I*–*V* trace was then subtracted offline from all 30 original traces. Next, apamin-/iberiotoxin-sensitive currents were isolated by offline subtraction of *I*–*V* traces (mean of respective traces #14–#30) from average Ca^2+^-activated controls recorded previously in the same VSN. For the resulting *I*–*V* traces to be classified as either apamin- or iberiotoxin-sensitive Ca^2+^–activated K^+^ currents, two criteria must be met: (1) at membrane potentials (*V*_mem_) = −100 mV, negative inward currents must exceed −1.54 pA/pF, and (2) at *V*_mem_ = −70 mV, currents must be positive (i.e., current must reverse at *V*_mem_ < −70 mV). The negative current threshold (−1.54 pA/pF) was determined by calculating the mean − SD (−0.31 to 1.23 pA/pF) at *V*_mem_ = −100 mV from *I*–*V* traces isolated by subtraction when drugs (apamin or iberiotoxin) were omitted. Reversal potential (*V*_rev_) values were calculated as the mean voltages at 0 ± 0.5 pA current.

For calculation of *V*_mem_ plateau potentials during positive current injections in *I*/*f* recordings, we plotted *V*_mem_ all-point histograms for values between maximum AP repolarization and firing threshold. Applying a Gaussian mixture model, the peak of the largest Gaussian distribution represents the *V*_mem_ plateau potential.

For analysis of single AP waveforms, we first isolated *V*_mem_ segments during positive current injections (30 ms/cycle) and applied a digital 1 kHz low-pass Butterworth filter to remove excess noise. Average waveforms of five consecutive APs (#1–5, #51–55, #96–100, #151–155) were calculated and represented as phase-plane (dV/dt) plots for unbiased automated extraction of AP waveform features (e.g., peak or repolarization potential).

#### Ca^2+^ imaging

Mean intensity values from individual ROIs were calculated in FIJI/ImageJ. After offset subtraction (background ROI), Ca^2+^-dependent changes in Cal-520 intensity were measured as the normalized fluorescence signal Δ*F*/*F*_0_, with (1) *F*_0_ corresponding to the average baseline fluorescence during the initial 3–20 frames of each recording (depending on experimental protocol) and (2) Δ*F* corresponding to the difference between the fluorescence intensity in a given frame and *F*_0_.

## Results

### VSN soma, dendrite, and knob are largely independent Ca^2+^ signaling compartments

**Figure 1.**
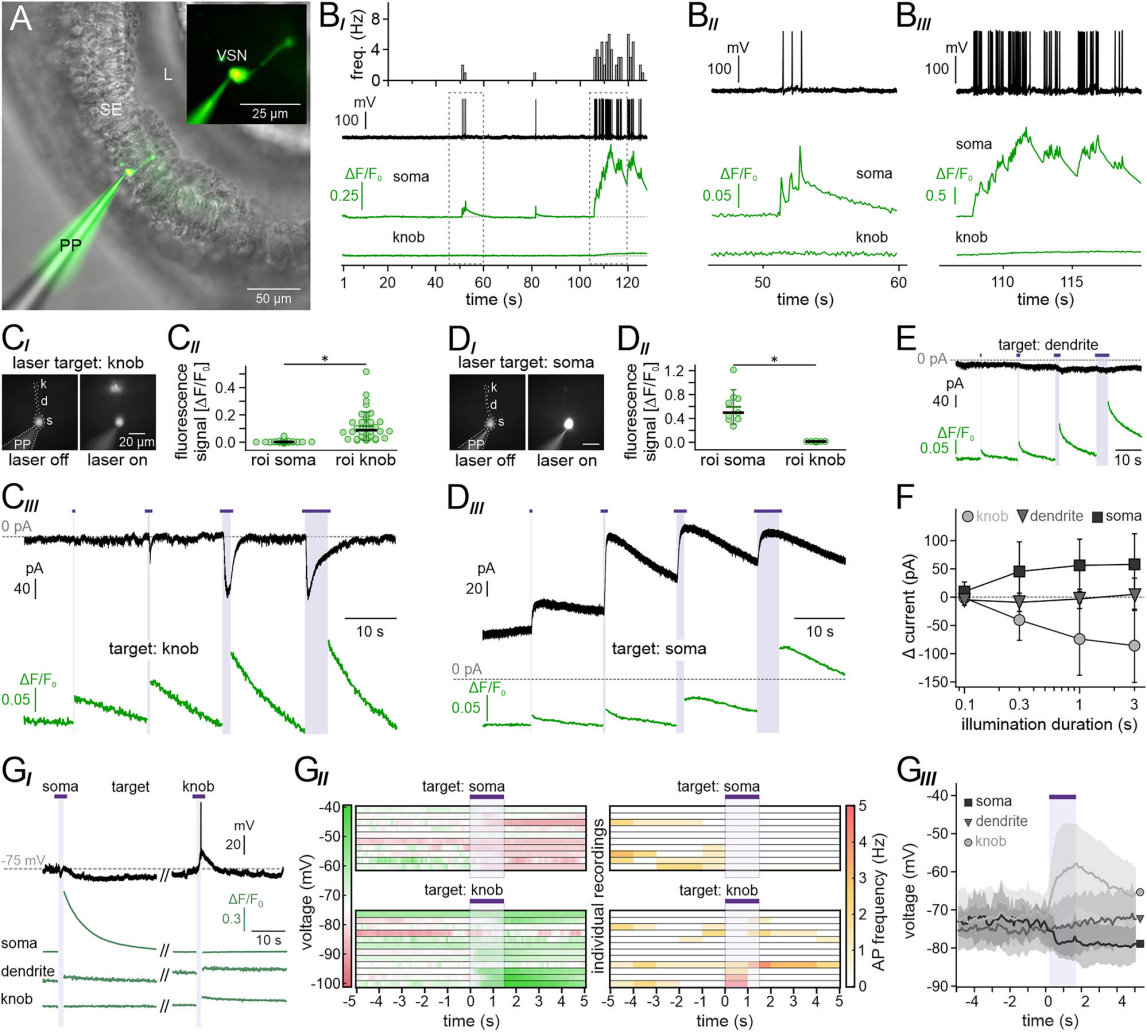
VSN soma and knob are independent Ca^2+^ signaling compartments. ***A***, Experiment configuration. Merged infrared differential interference contrast and epi-fluorescence image of an acute coronal VNO slice (150 μm). A VSN is targeted by a patch pipette (PP) and diffusion loaded with the Ca^2+^ indicator Cal-520 in whole-cell configuration. Lumen (L) and sensory epithelium (SE) as indicated. Inset, Fluorescence image outlining individual VSN morphology. ***B***, Simultaneous original recordings of membrane potential (*V*_mem_; black) and cytosolic Ca^2+^ concentration (green) in the VSN soma (top) and knob (bottom), respectively. ***B_I_***, Spontaneous firing plotted as frequency histogram (top). Periods outlined by dashed rectangles are depicted at increased temporal resolution in ***B_II_*** and ***B_III_***. ***C***, ***D***, Targeted photorelease of Ca^2+^ in the VSN knob (***C_I_***) and soma (***D_I_***), respectively, triggers Ca^2+^ elevations restricted to the targeted compartment (***C_II_***; knob, 0.12 ± 0.1; median = 0.09; *n* = 40; soma, 0.01 ± 0.01; median = 0.001; *n* = 36; ***D_II_***; knob, 0.0 ± 0.004; median = 0.001; *n* = 9; soma, 0.59 ± 0.29; median = 0.49; *n* = 9). Green horizontal bars, means ± SD; black bar, median. Asterisks denote statistical significance (*U* = 28; *p* = 3.3 × 10^−16^ (***C_II_***); *U* = 0; *p* = 6.48 × 10^−5^ (***D_II_***); Mann–Whitney *U* test). ***C_III_***, ***D_III_***, Simultaneous whole-cell voltage–clamp (*V*_hold_ = −60 mV; black) and Cal-520 fluorescence (green) recordings upon photostimulation at increasing durations (0.1, 0.3, 1, 3 s; purple horizontal bars) reveal robust inward (***C_III_***) or outward (***D_III_***) currents that coincide with spatially confined Ca^2+^ signals in knob (***C_III_***) and soma (***D_III_***), respectively. ***E***, Simultaneous voltage-clamp (*V*_hold_ = −60 mV; black) and Ca^2+^ (green) recordings upon dendritic photostimulation at increasing durations (purple horizontal bars). ***F***, Quantification of peak photorelease-dependent currents (corresponding to examples shown in ***C–E***) as a function of illumination period and targeted compartment. Photostimulation at soma (rectangles; mean ± SD): 0.1 s, 10.1 ± 16.7 pA (*n* = 5); 0.3 s, 45.5 ± 52.3 pA (*n* = 5); 1 s, 56.3 ± 46.3 pA (*n* = 7); 3 s, 58.1 ± 54.2 pA (*n* = 5). Photostimulation at dendrite (triangles; mean ± SD): 0.1 s, −4.7 ± 9.4 pA (*n* = 4); 0.3 s, −9.2 ± 16.2 pA (*n* = 4); 1 s, −3.1 ± 17.0 pA (*n* = 8); 3 s, 5.1 ± 28.6 pA (*n* = 4). Photostimulation at knob (circles; mean ± SD): 0.1 s, −1.5 ± 4.2 pA (*n* = 8); 0.3 s, −40.4 ± 36.0 pA (*n* = 8); 1 s, −74.0 ± 64.2 pA (*n* = 10); 3 s, −85.7 ± 64.6 pA (*n* = 8). ***G***, Effects of compartment-specific Ca^2+^ release on *V*_mem_. ***G_I_***, Representative traces from simultaneous recordings of *V*_mem_ (black) and Ca^2+^ signal (green) in VSN soma (top), dendrite (middle), and knob (bottom), respectively, upon photostimulation (purple horizontal bars) targeting the soma (left) or knob (right). ***G_II_***, Peristimulus heat maps depicting *V*_mem_ (left; 100 ms bins) or AP frequency (right; 1 s bins) of individual VSNs (rows) before, during, and after targeted Ca^2+^ photorelease (purple bars) in the soma (top) or knob (bottom), respectively. ***G_III_***, Average peristimulus *V*_mem_ recordings representing photostimulation at the soma (black; *n* = 10), dendrite (dark gray; *n* = 11), and knob (light gray; *n* = 12). Solid lines depict mean values; shadows indicate SD.

In addition to the electrical events associated with vomeronasal signal transduction, VSN signaling involves a significant biochemical component, i.e., the dynamic mobilization of cytosolic Ca^2+^ across broad spatial and temporal scales (Mohrhardt et al., 2018). Here, we used whole-cell patch–clamp recordings from VSNs in acute coronal tissue slices (Fig. 1*A*) to simultaneously monitor spontaneous AP discharges and concurrent changes in cytosolic Ca^2+^ concentration in soma, dendrite, and knob, respectively (Fig. 1*B*). While single APs evoked distinct Ca^2+^ transients in the soma, discharge-correlated Ca^2+^ elevations in the knob only occurred upon burst firing, likely a result of repeated spontaneous receptor current fluctuations as similarly observed in olfactory sensory neurons (Reisert, 2010). Next, we asked whether massive local Ca^2+^ signals spread from knob-to-soma or vice versa. Targeted photorelease of Ca^2+^ (Ellis-Davies, 2007) in either knob or soma triggered robust local Ca^2+^ elevations, which however did not spread beyond the confines of the targeted cellular compartment (Fig. 1*C*,*D*). These data demonstrate that VSN knob and soma function as largely independent Ca^2+^ signaling compartments.

Voltage-clamp recordings during targeted Ca^2+^ release revealed electrical responses that drastically differed depending on the release site. In the VSN knob, Ca^2+^ transients induced robust inward currents (Fig. 1*C_III_*), reminiscent of previously recorded Ca^2+^-activated Cl^−^ currents (Dibattista et al., 2012). In the soma, however, Ca^2+^ release triggered strong outward currents (Fig. 1*D_III_*). Notably, essentially no currents were evoked when photorelease was targeted to the dendrite (Fig. 1*E*,*F*). Given these spatially defined electrophysiological signatures, we next asked how compartment-dependent Ca^2+^ release affects VSN membrane potential. Targeted photorelease of Ca^2+^ in soma versus knob had opposite effects (Fig. 1*G_I_*). While Ca^2+^ elevations in the soma mediated lasting hyperpolarizations, Ca^2+^ release in the knob strongly depolarized most VSNs (Fig. 1*G_II_*). Some suprathreshold depolarizations triggered AP firing, whereas photorelease in the soma frequently silenced spontaneous activity. Again, no such effects were observed upon dendritic Ca^2+^ release (Fig. 1*G_III_*). Together, these results show that VSN knob and soma are two biochemically separated Ca^2+^ signaling compartments, in which unique Ca^2+^-activated conductances play somewhat opposite roles in VSN physiology.

### A large VSN subpopulation expresses Ca^2+^-activated ion channels at the soma

**Figure 2.**
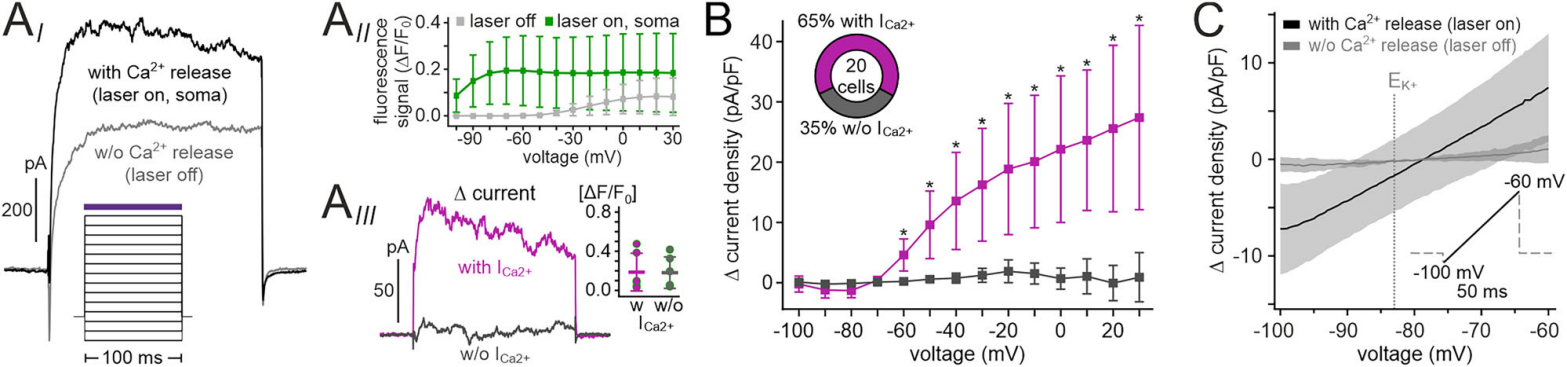
A large VSN subpopulation expresses Ca^2+^-activated ion channels at the soma. ***A***, ***B***, Isolation of Ca^2+^-activated currents upon soma-targeted Ca^2+^ release during step *I*–*V* recordings. ***A_I_***, Voltage-clamp pulse protocol and representative original traces showing currents evoked by depolarization (*V*_clamp_ = + 30 mV) both under control conditions (laser off; gray trace) and during photorelease of Ca^2+^ at the VSN soma (laser on, purple horizontal bar; black trace). ***A_II_***, Simultaneous imaging of cytosolic Cal-520 fluorescence confirmed robust Ca^2+^ elevations (green values) during all voltage steps. In the absence of photostimulation (gray values), depolarization ≥−20 mV also evoked Ca^2+^ signals, likely via opening of voltage-gated Ca^2+^ channels. ***A_III_***, Offline subtraction of control currents (laser off) isolates a Ca^2+^-dependent current (pink trace) or lack thereof (black trace). Note that photostimulation-evoked cytosolic Ca^2+^ elevations were confirmed in all VSNs, independent of the occurrence of a Ca^2+^-activated current (inset; *n* = 6, pink; *n* = 5, black). ***B***, Stepwise *I*–*V* relationship of isolated currents as shown in ***A_III_***. Of 20 VSNs examined, 13 neurons (65%) displayed a Ca^2+^-dependent current, whereas seven VSNs (35%) lacked such current. Asterisks indicate statistical significance (range, *U* = 2, *p* = 1.38 × 10^−4^ to *U* = 0, *p* = 5.19 × 10^−5^; Mann–Whitney *U* test). ***C***, Isolated average (mean ± SD, shadows; *n* = 7) upward voltage ramp recordings from VSNs under control conditions (laser off; gray) and during soma-targeted Ca^2+^ photorelease (black). The linear *I*–*V* relationship displays a reversal potential at −78.6 mV, close to the K^+^ equilibrium potential (*E*_K_; dotted vertical line).

While the photorelease-dependent inward current recorded in the knob most likely reflects Ca^2+^-dependent signal amplification via the TMEM16A channel (Yang and Delay, 2010; Kim et al., 2011; Dibattista et al., 2012; Amjad et al., 2015; Münch et al., 2018), the molecular effectors and physiological consequences of AP-dependent Ca^2+^ signals in the soma have rarely been addressed (and, in those rare cases, with contradictory results; Ukhanov et al., 2007; Zhang et al., 2008; Kim et al., 2012). Therefore, we analyzed the nature of the Ca^2+^-activated current(s) in VSN somata. First, we asked (1) if soma-targeted Ca^2+^ release activates currents in all VSNs and (2) whether these currents are voltage-dependent. In voltage-clamp recordings, stepwise hyper-/depolarization before and during photorelease revealed that Ca^2+^-activated currents occurred in the majority of VSNs (65%; Fig. 2*A*,*B*). Current activation appeared only weakly dependent on voltage, and absence of currents was not caused by insufficient Ca^2+^ release (Fig. 2*A_III_*). When measuring *I*–*V* curves in voltage ramp recordings, average photorelease-dependent currents displayed a roughly linear relationship at negative *V*_mem_ and reversed at approximately −79 mV, close to the K^+^ equilibrium potential (*E*_K_ = −83 mV; Fig. 2*C*). Together, these data demonstrate that most, but not all, VSNs express Ca^2+^-activated ion channels at their soma and that at least a substantial fraction of these are Ca^2+^-activated K^+^ channels.

### Somatic Ca^2+^ elevations activate SK and/or BK channels in distinct, yet partly overlapping VSN subpopulations

**Figure 3.**
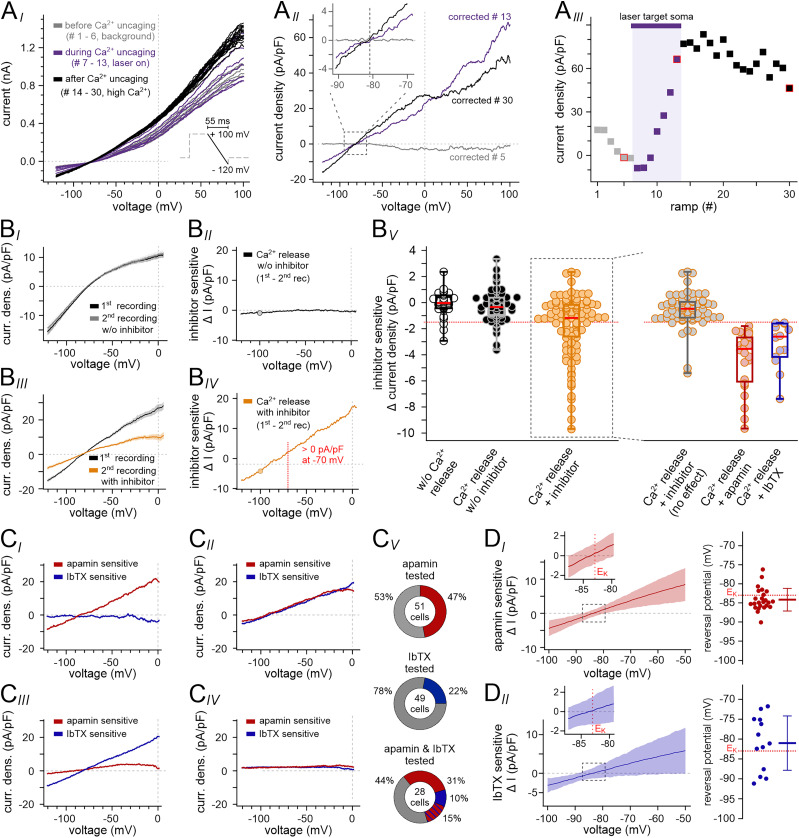
Somatic Ca^2+^ elevations activate SK and/or BK channels in distinct, yet partly overlapping VSN subpopulations. ***A***, Isolation of Ca^2+^ release-dependent *I*–*V* curves from a single representative VSN. ***A_I_***, Whole-cell VSN current traces from 30 consecutive downward voltage ramp recordings (inset, voltage-clamp protocol) before (#1–6; gray), during (#7–13; purple), and after (#14–30; black) soma-targeted photostimulation. ***A_II_***, Isolation of Ca^2+^-activated currents from three representative recordings (trace #5, #13, and #30) by offline subtraction of “background” current (average of traces #4–6). Inset details currents over the −90 to −70 mV (dashed rectangle) voltage range at higher magnification. *E*_K_ as indicated (dotted vertical line). ***A_III_***, Plot of current density measurements at +100 mV over time/ramp number. Color code as in ***A_I_***. Values corresponding to the three traces shown in ***A_II_*** are indicated by red edging. Photostimulation period indicated by the purple horizontal bar. ***B***, Strategy for isolation of drug-sensitive currents. ***B_I_***, *I*–*V* curves of Ca^2+^-activated currents are isolated as shown in ***A*** and depicted as mean traces (#14–30) plus SD (gray shadows). Average traces from two consecutive recording series under control conditions are overlaid. Note that the *I*–*V* relationships of the first (black) and second (gray) series are essentially indistinguishable. Accordingly, subtraction of the second from the first trace (***B_II_***) fails to isolate substantial current. ***B_III_***, Pharmacological treatment, however, can result in diminished current, which is isolated upon subtraction (***B_IV_***) and characterized by negative current density at −100 mV (filled orange circle) and positive values at −70 mV (dotted vertical red line). ***B_V_***, Dot plots of isolated current densities (first–second recording) at −100 mV under different experimental conditions: (left) control recordings without photostimulation (black circles), Ca^2+^ photorelease without pharmacological treatment (filled black dots), and Ca^2+^ photorelease ± drug treatment (filled orange circles); (right) categorization of results from drug treatment: VSNs are classified as either not sensitive to treatment (gray) or sensitive to apamin (red; 100 nM) or iberiotoxin (IbTX; blue; 100 nM), respectively. Box-and-whisker plots with boxes representing the first-to-third quartiles. Median indicated by red horizontal bars (left-to-right, −0.04 pA/pF, −0.3 pA/pF, −1.16 pA/pF, −0.47 pA/pF, −3.55 pA/pF, −2.61 pA/pF). To be classified as an apamin-/IbTX-sensitive VSN, negative current densities must exceed −1.54 pA/pF (corresponding to the mean − SD value without drug treatment; dotted red horizontal line) and reverse at *V*_mem_ < −70 mV (***B_IV_***). ***C***, Distribution of apamin- and/or IbTX-sensitive currents among VSNs. ***C_I_–_IV_***, *I*–*V* curves of drug-sensitive Ca^2+^–activated currents isolated as shown in ***B_IV_***. VSNs either display apamin-sensitive currents, but lack IbTX-sensitive activity (***C_I_***), express both apamin- and IbTX-sensitive currents (***C_II_***), only show IbTX-sensitive currents (***C_III_***), or lack any such currents (***C_IV_***). ***C_V_***, Quantification of electrophysiological phenotypes. When tested for apamin-sensitive putative SK currents, 47% (24/51) of VSNs displayed a corresponding current profile (top). IbTX-sensitive putative BK currents were recorded in 22% (13/59) of neurons (middle). Out of 48 VSNs that were sequentially exposed to both apamin and IbTX, 15 neurons (31%; ***C_I_***) exclusively displayed putative SK currents, whereas 5 VSNs (10%; ***C_III_***) showed only putative BK currents. Seven neurons (15%; ***C_II_***) were sensitive to both apamin and IbTX, while 21 VSNs (44%; ***C_IV_***) were not affected by either treatment (bottom). ***D***, Left, Average *I*–*V* curves (mean ± SD) of apamin-sensitive (*n* = 24; ***D_I_***) or IbTX-sensitive (*n* = 13; ***D_II_***) Ca^2+^-activated currents. Insets detail currents over the −87 to −78 mV range (dashed rectangles) at higher magnification. *E*_K_ as indicated (dotted vertical line). Right, Dot plots displaying individual reversal potentials from all measured VSNs expressing either putative SK currents (red; −84.1 ± 3.0 mV; ***D_I_***) or putative BK currents (blue; −81.0 ± 6.8 mV; ***D_II_***).

We next aimed to identify the molecular correlate(s) of VSN Ca^2+^-activated K^+^ currents. To this end, we first isolated Ca^2+^ release-dependent *I*–*V* curves from consecutive downward voltage ramp recordings after soma-targeted photorelease (Fig. 3*A*). Next, we employed highly selective peptide inhibitors of specific Ca^2+^-activated K^+^ channels, i.e., apamin or iberiotoxin (IbTX), to isolate any drug-sensitive current components (Fig. 3*B*). When, under control conditions, Ca^2+^ release-dependent average traces from two consecutive recording series are subtracted from one another, essentially no current is isolated (Fig. 3*B_I,II_*). Subtraction after pharmacological treatment, however, can isolate substantial drug-sensitive current (Fig. 3*B_III,IV_*). While SK channels are the sole known targets of the honey bee toxin apamin (Berkefeld et al., 2010), the scorpion venom peptide IbTX selectively blocks the BK channel pore with nanomolar affinity (Contreras et al., 2013). Exposure to either neurotoxin revealed both apamin- and IbTX-sensitive currents, respectively (Fig. 3*C*), with similar and relatively small current densities at *V*_mem_ = −100 mV (Fig. 3*B_V_*). We observed apamin-sensitive, putative SK currents in roughly half of all VSNs, whereas IbTX-sensitive, putative BK currents were recorded in 22% of neurons (Fig. 3*C*). Notably, when VSN were sequentially exposed to both drugs, these proportions were essentially confirmed, and we also found a fraction of neurons (15%) that displayed both SK and BK currents (Fig. 3*C_V_*). Average *I*–*V* curves of both apamin- and IbTX-sensitive currents as well as quantitative analysis of single *I*–*V* curve reversal potentials (Fig. 3*D*) confirm that both currents are carried by K^+^. Together these findings suggest that somatic Ca^2+^ elevations activate SK and/or BK channels in distinct, yet partly overlapping VSN subpopulations.

### A distinct VSN subpopulation displays putative Cl^−^ currents upon Ca^2+^ elevation in the soma

**Figure 4.**
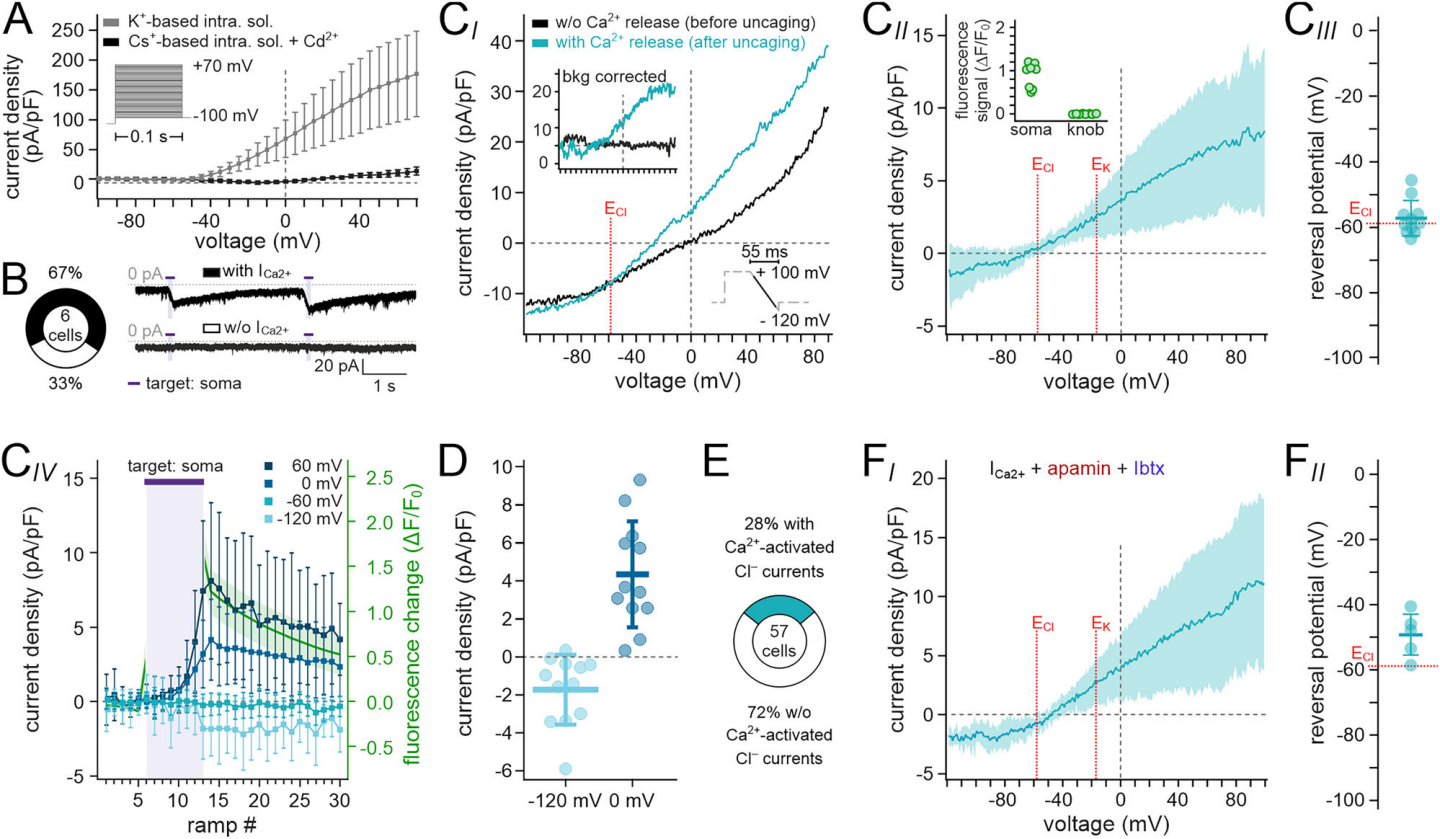
A distinct VSN subpopulation displays putative Cl^−^ currents upon Ca^2+^ elevation in the soma. ***A***, Voltage-clamp pulse protocol and corresponding stepwise *I*–*V* relationship of VSN steady-state outward currents recorded with either standard pipette solution (S_3_; gray; *n* = 19) or Cs^+^-based pipette solution (S_5_; black; *n* = 15) in the presence of extracellular Cd^2+^ (0.2 mM). ***B***, Whole-cell voltage–clamp recordings (S_5_ + Cd^2+^; *V*_hold_ = −75 mV) reveal transient inward currents upon repeated soma-targeted photostimulation (1 s; purple horizontal bars) in four out of six VSNs. ***C***, Isolation of Ca^2+^ release-dependent putative Cl^−^ currents. ***C_I_***, Thirty consecutive downward voltage ramp recordings (bottom inset, voltage-clamp protocol) allow isolation of whole-cell VSN *I*–*V* traces before (#6) and immediately after (#14) soma-targeted photostimulation (experimental paradigm as in Fig. 3*A*). Chloride equilibrium potential (*E*_Cl_) as indicated (dotted red vertical line). After offline subtraction of “background” current (average of traces #4–6), Ca^2+^-dependent currents become apparent (top inset). ***C_II_***, The average *I*–*V* curve (mean ± SD) of Ca^2+^-activated currents as recorded in **C*_I_***. *E*_K_ (−18 mV; 10 mM K^+^ in S_5_) and *E*_Cl_ (−59 mV) as indicated (dotted red vertical lines). Inset, While targeted photostimulation triggers robust Ca^2+^ elevations in the soma, no such signals are recorded from the knob (*n* = 8). ***C_III_***, The dot plot displaying individual reversal potentials (*n* = 11; −57.16 ± 5.36 mV; mean ± SD). *E*_Cl_ (−59 mV) as indicated. ***C_IV_***, Plot of current density measurements (*n* = 12; mean ± SD) at different membrane potentials (*V*_hold_ = −120, −60, 0, and 60 mV, respectively) over time/ramp number. Soma-targeted photostimulation period indicated by purple horizontal bar. Resulting changes in Ca^2+^ concentration are indicated by Cal-520 fluorescence (Δ*F*/*F*_0_; green). ***D***, Dot plots displaying peak current density measurements (ramp #14 or #15; *n* = 12) at −120 mV (left; −1.83 ± 1.8 pA/pF; mean ± SD) and 0 mV (right; 4.12 ± 2.73 pA/pF; mean ± SD), respectively. ***E***, Frequency of putative Ca^2+^-activated Cl^−^ currents in VSNs (16/57; 28%) upon soma-targeted Ca^2+^ elevation. ***F_I_***, Average *I*–*V* curve (means of ramps #12–16; *n* = 5 VSNs; mean ± SD) of Ca^2+^-activated currents recorded in the presence of both apamin and IbTX (100 nM each). *E*_K_ (−18 mV; 10 mM K^+^ in S_5_) and *E*_Cl_ (−59 mV) as indicated (dotted red vertical lines). ***F_II_***, Dot plot displaying individual reversal potentials (*n* = 5; −49.3 ± 7.02 mV; mean ± SD). *E*_Cl_ (−59 mV) as indicated.

Next, we asked whether additional non-K^+^ conductances are activated by local photorelease of Ca^2+^ in the VSN soma. Substitution of K^+^ by Cs^+^ in the pipette solution (S_5_) largely blocked K^+^ outward currents (Fig. 4*A*). Moreover, addition of extracellular Cd^2+^ inhibited Ca^2+^ influx via voltage-dependent Ca^2+^ channels. In sharp contrast to previous recordings (Fig. 1*D_III_*), when triggering Ca^2+^ transients in the VNS soma under these conditions, we induced relatively small, though robust inward currents in four out of six neurons (Fig. 4*B*). When again recording *I*–*V* curves from downward voltage ramps, the residual “background” current (i.e., without Ca^2+^ release) reversed at ∼0 mV (Fig. 4*C_I_*). In some VSNs, consecutive recordings after soma-targeted photorelease isolated a slightly outward rectifying current (Fig. 4*C_II_*) that reversed close to the Cl^−^ equilibrium potential (*E*_Cl_ = −58 mV; Fig. 4*C_III_*). Again, targeted UV illumination triggered robust Ca^2+^ elevations in the soma, whereas no Ca^2+^ signals were recorded in the knob (Fig. 4*C_II_*; inset). The decline of these putative Ca^2+^-activated Cl^−^ currents displayed strong correlation with the decay of the soma Ca^2+^ signal (Fig. 4*C_IV_*). Outward rectification became apparent in individual measurements (Fig. 4*D*). We observed such Ca^2+^-activated Cl^−^ currents in 28% of all VSNs (Fig. 4*E*). Notably, these currents remained essentially unaffected by addition of both apamin and IbTX (Fig. 4*F*). Together, these data demonstrate that a distinct VSN subpopulation expresses functional Ca^2+^-activated Cl^−^ channels at their soma. Thus, strong Ca^2+^ elevations in the VSN soma, as observed during AP discharge (Fig. 1*B*), will activate SK, BK, or Ca^2+^-activated Cl^−^ channels or various combinations thereof.

### SK channels modulate VSN discharge and dampen excitability

**Figure 5.**
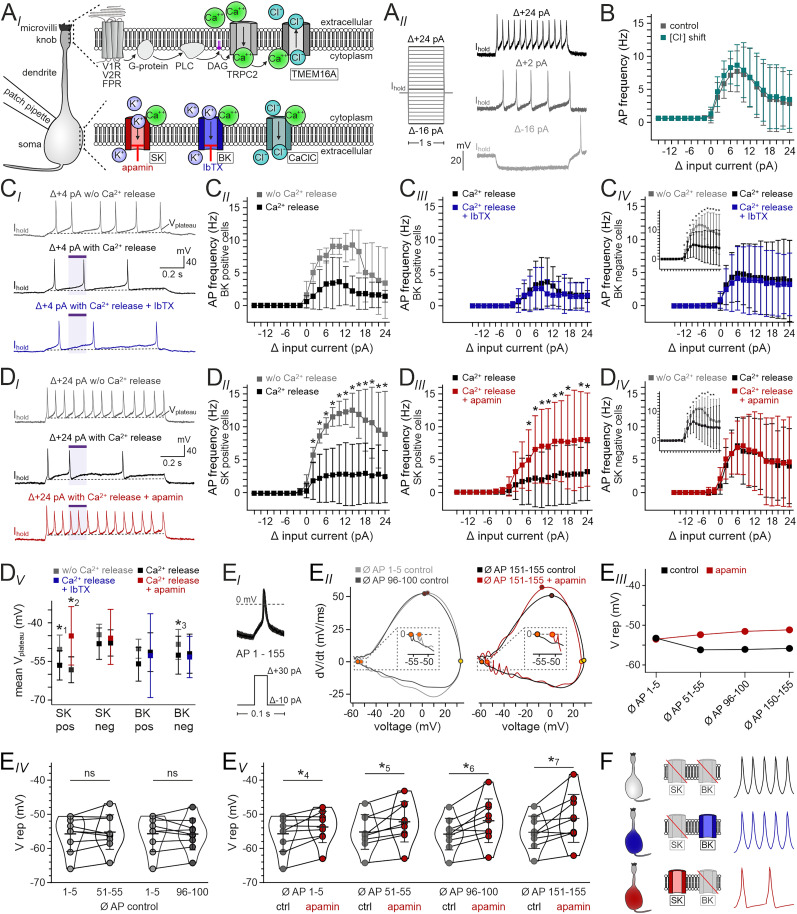
SK channels modulate VSN discharge and dampen excitability. ***A_I_***, Schematic illustrating the role of Ca^2+^ (green) in VSN primary signal transduction in the dendritic knob/microvilli (top) as well as in gating of Ca^2+^-activated K^+^ (SK and BK) and Cl^−^ channels (CaClC) in the VSN soma (bottom). SK channels (red) display high apamin affinity (*K*_D_ = 0.05–25 nM), whereas BK channels (blue) are selectively blocked by the African scorpion toxin IbTX. DAG, diacylglycerol; FPR, formyl peptide receptor; PLC, phospholipase C; V1/2R, vomeronasal type-1/-2 receptor. ***A_II_***, Representative current-clamp recordings of VSN membrane potential upon stepwise current injections (Δ−16 pA to Δ+24 pA; pulse protocol as indicated). Steady-state holding current (*I*_hold_) was selected to clamp neurons to approximately −75 mV. Few VSNs, particularly those with more hyperpolarized resting membrane potentials, displayed spontaneous activity in this voltage range. ***B***, Current injection/AP frequency plot (*I*/*f* curves). Data were recorded under control conditions (gray; *E*_Cl_ = −32.8 mV) and during incubation in reduced Cl^−^ extracellular solution (blue; *E*_Cl_ = −93.8 mV; *n* = 23). Shifting *E*_Cl_ did not affect the shape of the *I*/*f* curves (*p* > 0.05, Mann–Whitney *U* test). ***C***, ***D***, *I*/*f* recordings reveal effects of pharmacological treatment on VSN firing frequencies. ***C_I_***, ***D_I_***, Example voltage traces recorded upon depolarizing current injection under control conditions (gray), Ca^2+^ photorelease (black), and photostimulation in the presence of either IbTX (***C_I_***; blue) or apamin (***D_I_***; red). Photostimulation period indicated by purple horizontal bar; dashed horizontal lines indicate suprathreshold plateau potentials (*V*_plateu_). ***C_II_–_IV_***, ***D_II_–D_IV_***, Paired *I*/*f* curves recorded either under control conditions ± soma-targeted photostimulation (gray and black; ***C_II_*** and ***D_II_***; ***C_IV_*** and ***D_IV_***, inset) or upon Ca^2+^ photorelease ± IbTX (***C_III_*** and ***C_IV_***; blue; 100 nM) or apamin (***D_III_*** and ***D_IV_***; red; 100 nM). Based on previous voltage-clamp recordings, VSNs are classified as either expressing [***C_II_*** and ***C_III_*** (*n* = 5); ***D_II_*** and ***D_III_*** (*n* = 8)] or lacking [***C_IV_*** (*n* = 21) and ***D_IV_*** (*n* = 11)] somatic BK (***C***) or SK (***D***) channels. Asterisks indicate statistical significance [value ranges: *p* = 1.91 × 10^−6^ (*W* = 1) to 3.37 × 10^−2^ (*W* = 74.5; ***C_IV_***, inset); *p* = 7.81 × 10^−3^ (*W* = 0) to 3.13 × 10^−2^ (*W* = 2.5; ***D_II_***); *p* = 1.56 × 10^−2^ (*W* = 1) to 4.69 × 10^−2^ (*W* = 3.5; ***D_III_***); *p* = 1.95 × 10^−3^ (*W* = 0) to 4.69 × 10^−2^ (*W* = 5.5; ***D_IV_***, inset); Wilcoxon signed rank test]. Note that the relatively small fraction of BK-positive VSNs might underlie the lack of statistical significance in ***C_I_***. ***D_V_***, *V*_plateau_ (mean ± SD) recorded in VSNs classified as positive or negative for SK [left; *n* = 8 (pos), 11 (neg)] or BK [right; *n* = 5 (pos), 21 (neg)] channels. Colors indicate control conditions ± soma-targeted photostimulation (gray and black) or Ca^2+^ photorelease ± drug treatment (blue and red). Asterisks indicate statistical significance [*p*^1^ = 2.34 × 10^−2^ (*W* = 2); *p*^2^ = 1.56 × 10^−2^ (*W* = 1); *p*^3^ = 8.01 × 10^−3^ (*W* = 41); Wilcoxon signed ranks test]. ***E_I_***, Example current-clamp traces (top) of 200 overlaid APs fired in response to suprathreshold 30 ms somatic current injections at 10 Hz (pulse protocol; bottom). ***E_II_***, Phase-plane plots (dV/dt vs *V*_mem_; average of 5 APs each) of initial (1–5; gray), intermediate (96–100; black), and relatively late (151–155; black and red) APs. Maximum repolarization values (dV/dt = 0 mV/ms) are shown at higher magnification (dashed rectangles). Comparison of late APs reveals depolarized values upon apamin treatment (red; 100 nM). ***E_III_***, Example of maximum repolarization as a function of treatment (±apamin) and discharge duration. ***E_IV,V_***, Quantification of maximum repolarization (*V* rep) values over time under control conditions (***E_IV_***) as well as in the absence (gray) versus the presence (red) of apamin at different stages of prolonged firing (***E_V_***). Asterisks indicate statistical significance (*t*_(8)_ = 2.7, *p*^4^ = 2.52 × 10^−2^; *t*_(8)_ = 3.3, *p*^5^ = 1.05 × 10^−2^; *t*_(8)_ = 4.3, *p*^6^ = 2.45 × 10^−3^; *t*_(8)_ = 3.5, *p*^7^ = 8.06 × 10^−3^; paired Student’s *t* test); ns, not significant. ***F***, Schematic summary illustrating the three main fractions of VSNs as characterized by functional expression of Ca^2+^-activated K^+^ channels. Neurons either lack BK and SK channels (top) or express only BK (middle) or only SK (bottom). Note that, for clarity, the small population of VSNs that displays both putative BK and SK currents has been omitted. Drawings of example action potentials (right) describe the dampening effect of SK activity.

VSN activity is linked to pronounced Ca^2+^ signals in the soma (Figs. 1*B*, 5*A_I_*). Therefore, we next asked whether and, if so, how somatic Ca^2+^-activated Cl^−^ and/or K^+^ conductances affect VSN firing. To this end, we recorded current injection-dependent AP discharge (*I*/*f* curves; Fig. 5*A_II_*,*D*) in VSNs previously categorized as either expressing or lacking putative SK, BK, or Ca^2+^-activated Cl^−^ channels. Since highly specific drugs that selectively block Ca^2+^-activated Cl^−^ channels are currently missing (Alexander et al., 2023), we opted for shifting *E*_Cl_ during *I*/*f* recordings and asked whether this treatment affects basal firing properties. Perfusion with reduced Cl^−^ extracellular solution (S_1.2_ and S_2.2_) shifted *E*_Cl_ from −32.8 to −93.8 mV. This treatment, however, did not alter the characteristic shape of the *I*/*f* curve (Fig. 5*B*), indicating that Ca^2+^-activated Cl^−^ channels play a minor, if any, role for VSN high-frequency firing.

When comparing *I*/*f* curves recorded under control conditions with curves recorded during soma-targeted photorelease of Ca^2+^, we only observed a statistically significant reduction in firing frequency in VSNs previously categorized as lacking BK channels, not in those showing IbTX-sensitive K^+^ currents (Fig. 5*C_II,IV_*, inset). Accordingly, IbTX treatment did not affect firing properties during soma-targeted Ca^2+^ elevations in either VSN population (Fig. 5*C_III,IV_*). In contrast, a different picture emerged upon analyzing VSNs categorized as expressing versus lacking SK channels (Fig. 5*D*). Those neurons displaying apamin-sensitive K^+^ currents showed dramatically reduced firing frequencies upon Ca^2+^ photorelease (Fig. 5*D_I,II_*). Notably, this effect was rescued by apamin exposure (Fig. 5*D_I,III_*), whereas VSNs lacking putative SK channels showed no effect of apamin on Ca^2+^ release-dependent *I*/*f* curves (Fig. 5*D_IV_*). Corresponding effects were observed when analyzing plateau potentials underlying sustained trains of APs during strong depolarization (Fig. 5*C_I_*,*D_I,V_*). Here, upon Ca^2+^ photorelease, we recorded hyperpolarized plateau potentials exclusively in those VSNs either positive for SK or negative for BK channels. Notably, apamin reversed this effect only in neurons expressing putative SK channels (Fig. 5*D_V_*). Together, these data indicate that, in a specific VSN population, SK channel expression allows for Ca^2+^-dependent modulation of AP discharge.

Next, we asked whether SK channel expression affects the waveforms of individual APs upon repetitive high-frequency firing (Fig. 5*E*). When probed with 200 short suprathreshold current injections at 10 Hz (Fig. 5*E_I_*), VSNs reliably fired APs in response to each stimulation. Differentiation for dV/dt and associated phase-plane plot analysis of spike waveform (Fig. 5*E_II_*) revealed that apamin-dependent inhibition of SK channels results in a more depolarized maximum repolarization, an effect that appears to consolidate with increased firing duration (Fig. 5*E_III–V_*). No such effects were observed upon IbTX treatment (data not shown). Together, our results indicate that, within the subpopulation of SK channel expressing neurons, VSN discharge properties are modulated by somatic Ca^2+^ elevation and, consequently, activation of SK channels. Somatic SK recruitment hyperpolarizes the membrane and thus dampens excitability (Fig. 5*F*). This negative feedback mechanism could profoundly affect the VSN-to-AOB information transfer function.

## Discussion

Vomeronasal detection of conspecific chemosignals relies on both a complex signal transduction cascade in the VSN microvillous knob and transformation of that signal into AP discharge at the soma/axon. Information about the semiochemical environment is thus relayed to the AOB, the first center for processing of social and sexual cues in the brain. Both processes—signal transduction and AP generation—involve local Ca^2+^ elevations in the knob and soma, respectively. Here, we describe the compartmentalization of these Ca^2+^ signals, and we specifically address the electrophysiological impact of Ca^2+^ transients in the VSN soma.

The neural code underlying VSN-to-AOB information transfer is unclear. While the mechanisms by which neurons encode sensory information are diverse, ranging from spike rate integration and pattern recognition to individual spike timing (Rieke et al., 1997), neurons in the accessory olfactory system generally display rather slow dynamics (Yoles-Frenkel et al., 2018). Temporal constraints imposed by slow stimulus uptake and exchange via the vomeronasal pump (Meredith et al., 1980; Hamacher et al., 2024) likely determine prolonged stimulus-induced VSN responses (Luo et al., 2003; Ben-Shaul et al., 2010). Moreover, while reported stimulus-dependent spike frequency modulations range from ≤8 Hz (Kim et al., 2012; Chamero et al., 2017) up to 25–30 Hz (Stowers et al., 2002; Haga-Yamanaka et al., 2015), VSNs predominantly fire bursts of spikes at increased frequencies during stimulation (Arnson and Holy, 2011). Accordingly, prolonged burst firing upon sensory stimulation entails substantial Ca^2+^ entry into the VSN soma via voltage-gated channels (Liman and Corey, 1996; Fieni et al., 2003; Ackels et al., 2014). Somatic Ca^2+^-activated ion channels are thus ideally suited to endow VSNs with specific burst firing characteristics.

Ca^2+^ is a ubiquitous and probably the most multifaceted cellular messenger (Clapham, 2007). Its exact effects on VSN physiology are determined by the unique spatiotemporal profile of a given Ca^2+^ signal. Ca^2+^ elevations affect both primary and secondary signaling events and exert positive as well as negative feedback regulation (Chamero et al., 2012). Our findings indicate that cytoplasmic buffers, which limit Ca^2+^ diffusion, as well as extrusion and storage processes that rapidly restore resting conditions together generate local Ca^2+^ signaling domains in the VSN soma and knob, respectively. In such microdomains, Ca^2+^ concentrations rise up to 10 µM, with peak elevations in nanodomains (i.e., close to the site of release/influx) reaching 100 µM (Fakler and Adelman, 2008). In VSN dendritic knobs and microvilli, cytosolic Ca^2+^ elevations mainly result from TRPC2-mediated influx (Lucas et al., 2003) and maybe IP_3_-dependent store depletion (Yang and Delay, 2010; Kim et al., 2011) though the latter mechanism might be dispensable for primary chemoelectrical transduction (Chamero et al., 2017). Here, the main target for Ca^2+^-dependent signal amplification is a Ca^2+^-activated Cl^−^ channel, with TMEM16A forming its primary component (Dibattista et al., 2012; Amjad et al., 2015; Münch et al., 2018). Our recordings upon photolysis of caged Ca^2+^ in VSN knobs support these and other previous findings (Yang and Delay, 2010; Kim et al., 2011), although we do not rule out a contribution of Ca^2+^-activated cation channels (Liman, 2003; Spehr et al., 2009). In contrast, our data argue against a role of SK channels in primary signal transduction as has been previously proposed (Kim et al., 2012).

The physiological roles of Ca^2+^ signals in the VSN soma have been much less investigated. We have specifically addressed this issue by local photorelease of Ca^2+^ within the confines of the soma. Our results demonstrate that VSNs divide into subpopulations with distinct and only partially overlapping expression profiles of Ca^2+^-activated K^+^ channels. Approximately 50% of sensory neurons express functional SK channels, whereas a substantially smaller group displays BK channel activity. Both BK and SK currents are relatively small suggesting low expression density, especially for the large-conductance BK channel. However, given their extraordinarily high input resistance of several gigaohms (Liman and Corey, 1996; Shimazaki et al., 2006; Hagendorf et al., 2009), VSNs are exquisitely sensitive to electrical stimulation (Mohrhardt et al., 2018). First described in chromaffin and muscle cells (Marty, 1981; Pallotta et al., 1981), BK channels are widely expressed in neurons (Contreras et al., 2013). Gated by both voltage and cytoplasmic Ca^2+^, channel kinetics are inherently complex, especially during APs, where both voltage and the submembrane Ca^2+^ concentration change rapidly on a submillisecond timescale (Niday and Bean, 2021). In central neurons, BK channels were shown to contribute to AP repolarization, mediate the fast phase of the afterhyperpolarization, and shape dendritic Ca^2+^ spikes (Berkefeld et al., 2010). Previous work suggested that, by coupling to L-type Ca^2+^ channels, BK channels enable persistent VSN firing (Ukhanov et al., 2007). In contrast, others suggested a role in VSN sensory adaptation (Zhang et al., 2008). While both mechanisms could function in parallel, e.g., in different subcellular compartments (i.e., soma vs knob), our data suggest negligible BK channel activation upon Ca^2+^ elevation in the knob and no substantial involvement in discharge maintenance. Nonetheless, BK channels could play a role as a VSN “emergency brake” preventing cell damage or apoptosis under pathophysiological conditions that result in an extraordinarily large Ca^2+^ transient (Fakler and Adelman, 2008).

More prominent physiological functions in VSNs are exerted by SK channels. These voltage-independent channels are more sensitive to intracellular Ca^2+^ changes than BK is (Berkefeld et al., 2010) with *K*_D_ values of ≤1 µM. Thus, SK are activated by many Ca^2+^ sources and do not require such tight coupling to voltage-gated Ca^2+^ channels as BK channels do (Bond et al., 2005). SK deactivation is Ca^2+^-independent and occurs with time constants up to 60 ms (Xia et al., 1998; Pedarzani et al., 2001). These gating properties endow SK channels with a “short-term Ca^2+^ memory,” i.e., they remain active for >100 ms after Ca^2+^ resting levels are restored, thereby integrating even low-frequency Ca^2+^ signals over time (Berkefeld et al., 2010). Accordingly, neuronal SK channels have been implicated in intrinsic excitability and pacemaking (Wolfart et al., 2001), dendritic integration (Cai et al., 2004), and the slow afterhyperpolarization phase (Bond, 2004). A previous report has proposed an unconventional role of SK3 channels in VSN primary signal transduction (Kim et al., 2012). These authors show SK3 immunofluorescence in VSN cell bodies, dendrites, and dendritic knobs. In contrast, we do not observe putative K^+^ currents upon local Ca^2+^ release in knobs or dendrites. We do, however, record SK channel activity when Ca^2+^ is elevated in the soma. These K^+^ currents are apamin-sensitive, occur in approximately half of the VSN population, and limit repetitive firing frequency. It is tempting to speculate that SK expression could define two VSN classes according to different information transfer features. Future work will have to determine whether such functional dichotomy is reflected by known VSN subgroups (e.g., defined by receptor type expression). Together, our findings support a previous report of increased firing rates upon urine stimulation in the presence of apamin (Kim et al., 2012). Others, however, did not find any apamin effects on VSN firing, spike width, or interspike interval (Ukhanov et al., 2007).

Another intriguing observation is the functional expression of Ca^2+^-activated Cl^−^ channels in VSN somata. We recorded Cl^−^ currents upon soma-restricted Ca^2+^ release in 28% of VSNs. The identity and role (if any) of these channels in VSN physiology is currently unclear. It is possible that the channels merely represent TMEM16A proteins that are transported within the plasma membrane toward the dendritic tips. Likewise, they could just as well exert a different, yet to be identified function. So far, drastically shifting *E*_Cl_ from −32.8 to −93.8 mV did not affect VSN discharge parameters. This conclusion, however, is derived from a random sample of VSNs, not from the subgroup displaying Ca^2+^-activated Cl^−^ currents upon Ca^2+^ elevation in the soma. The specific experimental conditions required to isolate these currents in voltage-clamp experiments preclude classification prior to current-clamp recordings.

In summary, we demonstrate that mouse VSNs exhibit distinct and independent Ca^2+^ signaling compartments in their knobs, dendrites, and somata. Ca^2+^ elevations in knob and soma exert opposite effects. Ca^2+^ signals in the knob drive an excitatory inward current and, consequently, membrane depolarization and AP firing. In the soma, however, Ca^2+^ elevations hyperpolarize the membrane and thus dampen excitability in many VSNs. These effects are to a large extent mediated by SK channels. While not involved in dendritic signaling, these channels also modulate AP discharge and thus VSN-to-AOB information transfer.

## References

[B1] Ackels T, von der Weid B, Rodriguez I, Spehr M (2014) Physiological characterization of formyl peptide receptor expressing cells in the mouse vomeronasal organ. Front Neuroanat 8:134. 10.3389/fnana.2014.00134 25484858 PMC4240171

[B2] Alexander SPH, et al. (2023) The concise guide to PHARMACOLOGY 2023/24: ion channels. Br J Pharmacol 180:S145–S222. 10.1111/bph.16178 38123150 PMC11339754

[B3] Amjad A, Hernandez-Clavijo A, Pifferi S, Maurya DK, Boccaccio A, Franzot J, Rock JR, Menini A (2015) Conditional knockout of TMEM16A/anoctamin1 abolishes the calcium-activated chloride current in mouse vomeronasal sensory neurons. J Gen Physiol 145:285–301. 10.1085/jgp.201411348 25779870 PMC4380210

[B4] Arnson HA, Holy TE (2011) Chemosensory burst coding by mouse vomeronasal sensory neurons. J Neurophysiol 106:409–420. 10.1152/jn.00108.2011 21525370 PMC3129729

[B5] Barry PH (1984) Slow potential changes due to transport number effects in cells with unstirred membrane invaginations or dendrites. J Membr Biol 82:221–239. 10.1007/BF01871632 6099423

[B6] Ben-Shaul Y, Katz LC, Mooney R, Dulac C (2010) In vivo vomeronasal stimulation reveals sensory encoding of conspecific and allospecific cues by the mouse accessory olfactory bulb. Proc Natl Acad Sci U S A 107:5172–5177. 10.1073/pnas.0915147107 20194746 PMC2841925

[B7] Berkefeld H, Fakler B (2008) Repolarizing responses of BK _Ca_ –Cav complexes are distinctly shaped by their Cav subunits. J Neurosci 28:8238–8245. 10.1523/JNEUROSCI.2274-08.2008 18701686 PMC6670555

[B8] Berkefeld H, Fakler B, Schulte U (2010) Ca^2+^ -activated K^+^ channels: from protein complexes to function. Physiol Rev 90:1437–1459. 10.1152/physrev.00049.2009 20959620

[B9] Bond CT (2004) Small conductance Ca2+-activated K+ channel knock-out mice reveal the identity of calcium-dependent afterhyperpolarization currents. J Neurosci 24:5301–5306. 10.1523/JNEUROSCI.0182-04.2004 15190101 PMC2831645

[B10] Bond CT, Maylie J, Adelman JP (2005) SK channels in excitability, pacemaking and synaptic integration. Curr Opin Neurobiol 15:305–311. 10.1016/j.conb.2005.05.001 15922588

[B11] Cai X, Liang CW, Muralidharan S, Kao JPY, Tang C-M, Thompson SM (2004) Unique roles of SK and Kv4.2 potassium channels in dendritic integration. Neuron 44:351–364. 10.1016/j.neuron.2004.09.026 15473972

[B12] Chamero P, Leinders-Zufall T, Zufall F (2012) From genes to social communication: molecular sensing by the vomeronasal organ. Trends Neurosci 2:1–10. 10.1016/j.tins.2012.04.011 22658923

[B13] Chamero P, Marton TF, Logan DW, Flanagan KA, Cruz JR, Saghatelian A, Cravatt BF, Stowers L (2007) Identification of protein pheromones that promote aggressive behaviour. Nature 450:899–902. 10.1038/nature05997 18064011

[B14] Chamero P, Weiss J, Alonso MT, Rodríguez-Prados M, Hisatsune C, Mikoshiba K, Leinders-Zufall T, Zufall F (2017) Type 3 inositol 1,4,5-trisphosphate receptor is dispensable for sensory activation of the mammalian vomeronasal organ. Sci Rep 7:10260. 10.1038/s41598-017-09638-8 28860523 PMC5579292

[B15] Cichy A, et al. (2015) Extracellular pH regulates excitability of vomeronasal sensory neurons. J Neurosci 35:4025–4039. 10.1523/JNEUROSCI.2593-14.2015 25740530 PMC6605571

[B16] Clapham DE (2007) Calcium signaling. Cell 131:1047–1058. 10.1016/j.cell.2007.11.028 18083096

[B17] Contreras GF, et al. (2013) A BK (Slo1) channel journey from molecule to physiology. Channels 7:442–458. 10.4161/chan.26242 24025517 PMC4042479

[B18] Dibattista M, Amjad A, Maurya DK, Sagheddu C, Montani G, Tirindelli R, Menini A (2012) Calcium-activated chloride channels in the apical region of mouse vomeronasal sensory neurons. J Gen Physiol 140:3–15. 10.1085/jgp.201210780 22732308 PMC3382724

[B19] Ellis-Davies GCR (2007) Caged compounds: photorelease technology for control of cellular chemistry and physiology. Nat Methods 4:619–628. 10.1038/nmeth1072 17664946 PMC4207253

[B20] Fakler B, Adelman JP (2008) Control of KCa channels by calcium nano/microdomains. Neuron 59:873–881. 10.1016/j.neuron.2008.09.001 18817728

[B21] Fieni F, Ghiaroni V, Tirindelli R, Pietra P, Bigiani A (2003) Apical and basal neurones isolated from the mouse vomeronasal organ differ for voltage-dependent currents. J Physiol 552:425–436. 10.1113/jphysiol.2003.052035 14561826 PMC2343397

[B22] Haga-Yamanaka S, Ma L, Yu CR (2015) Tuning properties and dynamic range of type 1 vomeronasal receptors. Front Neurosci 9:244. 10.3389/fnins.2015.00244 26236183 PMC4501179

[B23] Haga S, Hattori T, Sato T, Sato K, Matsuda S, Kobayakawa R, Sakano H, Yoshihara Y, Kikusui T, Touhara K (2010) The male mouse pheromone ESP1 enhances female sexual receptive behaviour through a specific vomeronasal receptor. Nature 466:118–122. 10.1038/nature09142 20596023

[B24] Haga S, Hiroko K, Kazushige T (2007) Molecular characterization of vomeronasal sensory neurons responding to a male-specific peptide in tear fluid: sexual communication in mice. Pure Appl Chem 79:775. 10.1351/pac200779040775

[B25] Hagendorf S, Fluegge D, Engelhardt CH, Spehr M (2009) Homeostatic control of sensory output in basal vomeronasal neurons: activity-dependent expression of ether-à-go-go-related gene potassium channels. J Neurosci 29:206–221. 10.1523/JNEUROSCI.3656-08.2009 19129398 PMC6664915

[B26] Hamacher C, et al. (2024) A revised conceptual framework for mouse vomeronasal pumping and stimulus sampling. Curr Biol 34:1206–1221.e6. 10.1016/j.cub.2024.01.036 38320553 PMC10965388

[B27] Jacobson L, Trotier D, Døving KB (1998) Anatomical description of a new organ in the nose of domesticated animals by Ludvig Jacobson (1813). Chem Senses 23:743–754. 10.1093/chemse/23.6.743 9915121

[B28] Kelliher KR, Spehr M, Li X-HH, Zufall F, Leinders-Zufall T (2006) Pheromonal recognition memory induced by TRPC2-independent vomeronasal sensing. Eur J Neurosci 23:3385–3390. 10.1111/j.1460-9568.2006.04866.x 16820028

[B29] Kim S, Ma L, Jensen KL, Kim MM, Bond CT, Adelman JP, Yu CR (2012) Paradoxical contribution of SK3 and GIRK channels to the activation of mouse vomeronasal organ. Nat Neurosci 15:1236–1244. 10.1038/nn.3173 22842147 PMC3431453

[B30] Kim S, Ma L, Unruh J, McKinney S, Yu CR (2015) Intracellular chloride concentration of the mouse vomeronasal neuron. BMC Neurosci 16:90. 10.1186/s12868-015-0230-y 26667019 PMC4678706

[B31] Kim S, Ma L, Yu CR (2011) Requirement of calcium-activated chloride channels in the activation of mouse vomeronasal neurons. Nat Commun 2:365. 10.1038/ncomms1368 21694713 PMC3156823

[B32] Lê Cao KA, Boitard S, Besse P (2011) Sparse PLS discriminant analysis: biologically relevant feature selection and graphical displays for multiclass problems. BMC Bioinformatics 12:253. 10.1186/1471-2105-12-253 21693065 PMC3133555

[B33] Leinders-Zufall T, Brennan PA, Widmayer P, Prashanth CS, Maul-Pavicic A, Jäger M, Li X-H, Breer H, Zufall F, Boehm T (2004) MHC class I peptides as chemosensory signals in the vomeronasal organ. Science 306:1033–1037. 10.1126/science.1102818 15528444

[B34] Leinders-Zufall T, Lane AP, Puche AC, Ma W, Novotny MV, Shipley MT, Zufall F (2000) Ultrasensitive pheromone detection by mammalian vomeronasal neurons. Nature 405:792–796. 10.1038/35015572 10866200

[B35] Liman ER (2003) Regulation by voltage and adenine nucleotides of a Ca2+-activated cation channel from hamster vomeronasal sensory neurons. J Physiol 548:777–787. 10.1113/jphysiol.2002.037119 12640014 PMC2342889

[B36] Liman ER, Corey DP (1996) Electrophysiological characterization of chemosensory neurons from the mouse vomeronasal organ. J Neurosci 16:4625–4637. 10.1523/JNEUROSCI.16-15-04625.1996 8764651 PMC6579035

[B37] Liman ER, Corey DP, Dulac C (1999) TRP2: a candidate transduction channel for mammalian pheromone sensory signaling. Proc Natl Acad Sci U S A 96:5791–5796. 10.1073/pnas.96.10.5791 10318963 PMC21939

[B38] Lucas P, Ukhanov K, Leinders-Zufall T, Zufall F (2003) A diacylglycerol-gated cation channel in vomeronasal neuron dendrites is impaired in TRPC2 mutant mice: mechanism of pheromone transduction. Neuron 40:551–561. 10.1016/S0896-6273(03)00675-5 14642279

[B39] Luo M, Fee MS, Katz LC (2003) Encoding pheromonal signals in the accessory olfactory bulb of behaving mice. Science 299:1196–1201. 10.1126/science.1082133 12595684

[B40] Marty A (1981) Ca-dependent K channels with large unitary conductance in chromaffin cell membranes. Nature 291:497–500. 10.1038/291497a0 6262657

[B41] Meredith M (1991) Sensory processing in the main and accessory olfactory systems: comparisons and contrasts. J Steroid Biochem Mol Biol 39:601–614. 10.1016/0960-0760(91)90258-7 1892791

[B42] Meredith M, Marques DM, O’Connell RJ, Stern FL (1980) Vomeronasal pump: significance for male hamster sexual behavior. Science 207:1224–1226. 10.1126/science.7355286 7355286

[B43] Mohrhardt J, Nagel M, Fleck D, Ben-Shaul Y, Spehr M (2018) Signal detection and coding in the accessory olfactory system. Chem Senses 43:667–695. 10.1093/chemse/bjy061 30256909 PMC6211456

[B44] Münch J, Billig GM, Huebner CA, Leinders-Zufall T, Zufall F, Jentsch TJ (2018) Ca2+-activated Cl− currents in the murine vomeronasal organ enhance neuronal spiking but are dispensable for male-male aggression. J Biol Chem 293:10392–10403. 10.1074/jbc.RA118.003153 29769308 PMC6028972

[B45] Nagel M, Niestroj M, Bansal R, Fleck D, Lampert A, Stopkova R, Stopka P, Ben-Shaul Y, Spehr M (2024) Deciphering the chemical language of inbred and wild mouse conspecific scents. Elife 12:RP90529. 10.7554/eLife.90529 38747258 PMC11095937

[B46] Niday Z, Bean BP (2021) BK channel regulation of after-potentials and burst firing in cerebellar Purkinje neurons. J Neurosci 41:2854–2869. 10.1523/JNEUROSCI.0192-20.2021 33593855 PMC8018884

[B47] Nodari F, Hsu F-F, Fu X, Holekamp TF, Kao L-F, Turk J, Holy TE (2008) Sulfated steroids as natural ligands of mouse pheromone-sensing neurons. J Neurosci 28:6407–6418. 10.1523/JNEUROSCI.1425-08.2008 18562612 PMC2726112

[B48] Pallotta BS, Magleby KL, Barrett JN (1981) Single channel recordings of Ca^2+^-activated K+ currents in rat muscle cell culture. Nature 293:471–474. 10.1038/293471a0 6273730

[B49] Papes F, Logan DW, Stowers L (2010) The vomeronasal organ mediates interspecies defensive behaviors through detection of protein pheromone homologs. Cell 141:692–703. 10.1016/j.cell.2010.03.037 20478258 PMC2873972

[B50] Pedarzani P, Mosbacher J, Rivard A, Cingolani LA, Oliver D, Stocker M, Adelman JP, Fakler B (2001) Control of electrical activity in central neurons by modulating the gating of small conductance Ca2+-activated K+ channels. J Biol Chem 276:9762–9769. 10.1074/jbc.M010001200 11134030

[B51] Reisert J (2010) Origin of basal activity in mammalian olfactory receptor neurons. J Gen Physiol 136:529–540. 10.1085/jgp.201010528 20974772 PMC2964517

[B52] Rieke F, Warland D, De Ruyter Van Steveninck R, Bialek W (1997). Spikes: exploring the neural code (Sejnowski TJ, Poggio TA eds). Cambridge, Massachusetts: The MIT Press.

[B53] Rodriguez I, Feinstein P, Mombaerts P (1999) Variable patterns of axonal projections of sensory neurons in the mouse vomeronasal system. Cell 97:199–208. 10.1016/S0092-8674(00)80730-8 10219241

[B54] Shimazaki R, Boccaccio A, Mazzatenta A, Pinato G, Migliore M, Menini A (2006) Electrophysiological properties and modeling of murine vomeronasal sensory neurons in acute slice preparations. Chem Senses 31:425–435. 10.1093/chemse/bjj047 16547196

[B55] Spehr J, Hagendorf S, Weiss J, Spehr M, Leinders-Zufall T, Zufall F (2009) Ca^2+^-calmodulin feedback mediates sensory adaptation and inhibits pheromone-sensitive ion channels in the vomeronasal organ. J Neurosci 29:2125–2135. 10.1523/JNEUROSCI.5416-08.2009 19228965 PMC6666346

[B56] Spehr M, Hatt H, Wetzel CH (2002) Arachidonic acid plays a role in rat vomeronasal signal transduction. J Neurosci 22:8429–8437. 10.1523/JNEUROSCI.22-19-08429.2002 12351717 PMC6757791

[B57] Stowers L, Holy TE, Meister M, Dulac C, Koentges G (2002) Loss of sex discrimination and male-male aggression in mice deficient for TRP2. Science 295:1493–1500. 10.1126/science.1069259 11823606

[B58] Tirindelli R, Dibattista M, Pifferi S, Menini A (2009) From pheromones to behavior. Physiol Rev 89:921–956. 10.1152/physrev.00037.2008 19584317

[B59] Turaga D, Holy TE (2012) Organization of vomeronasal sensory coding revealed by fast volumetric calcium imaging. J Neurosci 32:1612–1621. 10.1523/JNEUROSCI.5339-11.2012 22302803 PMC3342671

[B60] Ukhanov K, Leinders-Zufall T, Zufall F (2007) Patch-clamp analysis of gene-targeted vomeronasal neurons expressing a defined V1r or V2r receptor: ionic mechanisms underlying persistent firing. J Neurophysiol 98:2357–2369. 10.1152/jn.00642.2007 17715188

[B61] Untiet V, Moeller LM, Ibarra-Soria X, Sánchez-Andrade G, Stricker M, Neuhaus EM, Logan DW, Gensch T, Spehr M (2016) Elevated cytosolic Cl-concentrations in dendritic knobs of mouse vomeronasal sensory neurons. Chem Senses 41:669–676. 10.1093/chemse/bjw077 27377750 PMC5030740

[B62] Veitinger S, et al. (2011) Purinergic signalling mobilizes mitochondrial Ca2+ in mouse Sertoli cells. J Physiol 589:5033–5055. 10.1113/jphysiol.2011.216309 21859825 PMC3225664

[B63] Wolfart J, Neuhoff H, Franz O, Roeper J (2001) Differential expression of the small-conductance, calcium-activated potassium channel SK3 is critical for pacemaker control in dopaminergic midbrain neurons. J Neurosci 21:3443–3456. 10.1523/JNEUROSCI.21-10-03443.2001 11331374 PMC6762487

[B64] Wong WM, Nagel M, Hernandez-Clavijo A, Pifferi S, Menini A, Spehr M, Meeks JP (2018) Sensory adaptation to chemical cues by vomeronasal sensory neurons. eNeuro 5:ENEURO.0223-18.2018. 10.1523/ENEURO.0223-18.2018 30105301 PMC6088365

[B65] Xia X-M, et al. (1998) Mechanism of calcium gating in small-conductance calcium-activated potassium channels. Nature 395:503–507. 10.1038/26758 9774106

[B66] Yang C, Delay RJ (2010) Calcium-activated chloride current amplifies the response to urine in mouse vomeronasal sensory neurons. J Gen Physiol 135:3–13. 10.1085/jgp.200910265 20038523 PMC2806418

[B67] Yoles-Frenkel M, Kahan A, Ben-Shaul Y (2018) Temporal response properties of accessory olfactory bulb neurons: limitations and opportunities for decoding. J Neurosci 38:4957–4976. 10.1523/JNEUROSCI.2091-17.2018 29712784 PMC6596120

[B68] Yu CR (2015) TRICK or TRP? What Trpc2-/- mice tell us about vomeronasal organ mediated innate behaviors. Front Neurosci 9:221. 10.3389/fnins.2015.00221 26157356 PMC4477137

[B69] Zhang P, Yang C, Delay RJ (2008) Urine stimulation activates BK channels in mouse vomeronasal neurons. J Neurophysiol 100:1824–1834. 10.1152/jn.90555.2008 18701755 PMC2576191

[B70] Zhang P, Yang C, Delay RJ (2010) Odors activate dual pathways, a TRPC2 and a AA-dependent pathway, in mouse vomeronasal neurons. Am J Physiol Cell Physiol 298:C1253–64. 10.1152/ajpcell.00271.2009 20147653 PMC2867386

